# Comparison urine neutrophil gelatinase - associated lipocalin with standard parameters in monitoring activity Lupus nephritis: Class IV

**DOI:** 10.5937/jomb0-35933

**Published:** 2023-01-20

**Authors:** Violeta Rabrenović, Milica Petrović, Milorad Rabrenović

**Affiliations:** 1 Military Medical Academy, Clinic of Nephrology, Belgrade; 2 University of Defense, Military Medical Academy, Faculty of Medicine, Belgrade; 3 Military Medical Academy, Center for Hyperbaric Medicine, Belgrade

**Keywords:** class IV lupus nephritis, activity, neutrophil gelatinase-associated lipocalin, proteinuria, lupus nefritis klasa IV, aktivnost, lipokalin udružen sa neutrofilnom gelatinazom, proteinurija

## Abstract

**Background:**

Lupus nephritis (LN) is one of the most serious complications in the development of systemic lupus erythematosus, that can adversely affect the course and prognosis of this autoimmune disease. Therefore, monitoring the effect of applied therapy, achieving remission, or monitoring class IV LN activity is still a great challenge for nephrologists. This study aimed to compare the urinary neutrophile gelatinase associated lipocalin (u/NGAL) with traditionally accepted parameters for LNactivity to indicate the importance of its determination in these patients.

**Methods:**

The study group consisted of 40 patients with class IV LN, who were prospectively followed for a period of 4 months within three control visits to 2 months. The first group (20/40) had active disease (Group A), and the second group had diseasein remission (Group B). The parameters we monitored and compared at each visit were standard biochemical parameters and kidney function parameters: C-reactive protein (CRP), blood count (CBC), creatinine, total proteins, albumin, cholesterol, triglycerides, glomerular filtration rate (eGFR). Regarding immune parameters, complement C3 and C4, antinuclear antibodies (ANA), anti-double stranded DNA antibody(anti ds DNA Ab) were monitored. Urine sediment, proteinuria 24h, urine culture, urinary protein/creatinine ratio - Up/Cre, and urinary NGAL (u/NGAL) were monitored in

**Results:**

Comparing standard parameters of disease activity and u/NGAL between groups, a statistically significant difference was obtained (p < 0.001). Within Group A, comparing the parameters by visits (0 : 2) for anti-ds-DNA Ab a significance of p< 0.05 was obtained, for albumin/s and C3 a significance of p<0.01 was obtained, and proteinuria/24h, Up/Cre, u/NGAL had a significance of p < 0.001. The mean level of u/NGAL was elevated at the initially visit (173.25 ± 172.12 ng/mL), after two months 73.2 ± 48.7 ng/mL, and in the second visit a lower level was recorded (49.60 ± 72.57 ng/mL). The negative correlation of u/NGAL was statistically significant at initial visit with albumin/s (p< 0.01) as well as the positive correlation with proteinuria 24h and Up/Cre (p< 0.001). In visit 2 significant negative correlation of u/NGAL with albumin/s and C3 p< 0.05, and positive correlation with anti-ds-DNA Ab, proteinuria 24h and Up/Cre p < 0.001.

**Conclusions:**

The results of our study indicate that the level of u/N GLA is elevated in patients with active Lupus nephritis class IV, as well as that it correlates with other parameters of disease activity. Serial determination of u/NGAL could be significant in monitoring disease course and treatment

## Abstract (sr)

**Uvod:**

Lupus nefritis (LN) je jedna od najozbiljnijih komplikacija u razvoju sistemskog eritemskog lupusa, koja može negativno uticati na tok i prognozu ove autoimune bolesti. Stoga je praćenje efekta primenjene terapije, postizanje remisije ili praćenje aktivnosti LN i dalje veliki izazov za nefrologe. Ova studija je imala za cilj da uporedi lipokalin udružen sa neutrofilnom gelatinazom u urinu (u/NGAL) sa tradicionalno prihvaćenim parametrima za aktivnost LN, kako bi se ukazalo na važnost njegovog određivanja kod ovih pacijenata.

**Metode:**

Studijsku grupu je činilo 40 pacijenata, sa LN klasa IV, koji su prospektivno praćeni u periodu od 4 meseca u okviru tri posete na 2 meseca. Prva grupa (20/40) imala je aktivni LN (Grupa A), a druga grupa imala je LN u remisiji (Grupa B). Parametri koji su praćeni i poređeni bili su standardni biohemijski parametri i parametri funkcije bubrega: C-reaktivni protein (CRP), krvna slika (CBC), kreatinin, ukupni proteini, albumin, holesterol, trigliceridi, brzina glomerulske filtracije (eGFR).Od imunskih parametara: komplement C3, C4, ANA, anti-ds-DNK At: U urinu: sediment, proteinurija 24h, odnos ur.protein/ kreatinin (Up/Cre), u/N GAL (imunohemijska metoda CM IA),GFR.

**Rezultati:**

Poređenjem standardnih parametara aktivnosti bolesti i u/NGAL između grupa, dobijena je statistički značajna razlika (p < 0 ,0 0 1 ). U okviru Grupe A, poređenjem parametara po posetama (0 : 2) za anti dsDNK At dobijena je značajnost p< 0,05, za albumine i C3 p< 0,05, a za proteinuriju /24h, U p/Cre i u/NGAL p< 0,001. Nivo u/NGAL je u prvoj poseti bio povišen (173,25 ± 172,12 ng/mL), nakon 2 meseca je iznosio 73,2 ± 48,7 ng/mL, a u drugoj viziti beleži se niža vrednost 49,60 ± 72,57 ng/mL. Negativna korelacija u/NGAL je bila statistički značajna u prvoj poseti sa albuminom p < 0,01, kao i pozitivna korelacija sa proteinurijom 24h i Up/Cre (p < 0,001). U poseti 2 nađena je značajna negativna korelacija u/NGAL sa albuminom i C3 (p < 0,05), i pozitivna korelacija sa anti ds-DNK At, proteinurijom 24h i Up/Cre (p< 0,001).

**Zaključak:**

Rezultati našeg istraživanja ukazuju da je nivo u/NGAL povišen kod pacijenata sa aktivnim LN klase IV, kao i da je u korelaciji sa drugim parametrima aktivnosti i lečenja bolesti. Serijsko određivanje u/NGAL moglo bi biti značajno u praćenju toka bolesti i lečenja.

## Introduction

The development of lupus nephritis (LN) in patients with systemic lupus erythematosus is a poor prognostic parameter. The treatment of these patients is long-term, frequent controls are necessary due to possible exacerbations of the disease.

A particularly severe form of LN is class IV lupus nephritis, which represents a diffusely proliferative form, with pronounced activity and rapid progression, and often with severe nephrotic syndrome and impairment kidney function. That is why the timely diagnosis of disease and initiation of treatment are of great importance. Previous standard biochemicalimmune parameters determining LN activity have shown limited utility, the manifestation of their activity often coincides with already significant kidney impairment [1] [2] [3] [4]. Kidney biopsy is still the standard for determining LN activity, but rebiopsies to monitor the further course and effect of treatment are not performed often [4] [5]. Neutrophil gelatinase-associated lipocalin (NGAL), also known as lipocalin 2, is a glycoprotein that binds to metalloproteinase 9 (MMP-9) in human neutrophils, and which is secreted from specific neutrophil granules after cell activation [6] [7]. So far, many studies examining NGAL have concluded that this marker is of multiple importance for acute kidney failure and as such should be routinely determined in intensive care units, in pediatrics, and surgery [8] [9] [10]. Numerous studies have described patients with acute kidney failure of various etiologies in whom the correlation of NGAL and serum creatinine levels has been confirmed [11] [12] [13] [14]. Chronic renal failure has also been shown to affect NGAL levels and may therefore be a limiting factor [15] [16]. This has also been confirmed in studies in patients with LN describing elevated levels of NGAL that correlate with disease activity,serum creatinine and impaired kidney function [17] [18]. This study aimed to invest in the importance of monitoring patients with class IV LN by determining u/NGAL and comparing them with standard parameters of disease activity and testing diagnostic accuracy.

## Materials and methods

The clinical-prospective study included 40 patients with systemic lupus erythematosus (SLE), which was confirmed by the criteria of the American College of Rheumatology (ACR) and LN which was confirmed by renal biopsy and histopathology findings (World Helth Organisation - WHO classification) [19] [20] [21] [22] [23]. The study included patients of both sexes and different ages (>18 years). Kidney disease activity was also classified according to the renal disease activity index : Systemic Lupus Erythematosus Disease Activity Index/renal (SLEDAI/r) [24]. SLEDAI/r consists of 4 criteria that grade renal impairment within the SLEDAI 2000 (Systemic Lupus Erythematosus Disease Activity Index-SLEDAI 2000) criteria of SLE activity [24].

*Including criteria* for the study: The first group (consisted of 20 patients) had active disease (Group A) which according to standard analysis was defined as proteinuria ≥ 0.5 g/24h; urinary protein/creatinine ratio(Up/Cre) >0.5; according to SLEDAI/r criteria, hypocomplementemia C3,C4, positive anti-double stranded DNA antibodies (anti ds DNA Ab)and patho-histological findings of renal biopsy. All patients had a glomerular filtration rate (eGFR) ≥ of 60 mL/min/1.73 m^2^ according to the Chronic Kidney Disease Epidemiology Collaboration (CKD-EPI). The second group (Group B): consisted of 20 patients with class IV LN who were in complete remission (according to the criterion: proteinuria ≤ 0.5g/24h., Up/Cre <0.5; negative anti ds DNA Ab, complement C3 and C4 within the reference range, and eGFR 60 mL/min/1.73 m^2^). *Excluding criteria* were the same for both groups: patients with urinary tract infection (positive urine culture), with renal calculosis, with kidney failure (CKDeGFR <60 mL/min/1.73 m^2^).The ratio of parameters at the beginning of treatment and during the expected changes in disease activity after 2 and 4 months after the treatment started was monitored. The parameters we monitored were standard biochemical parameters and kidney function parameters: C-reactive protein (CRP), blood count (CBC), creatinine, total proteins, albumin, cholesterol, triglycerides, eGFR. Regarding immune parameters, complement C3 and C4, antinuclear antibodies (ANA), anti ds DNA Ab were monitored. Urine sediment, proteinuria 24h (proteins from 24 hours collected urine), urine culture, Up/Cre were monitored in first morning urine prepared by the standard method.Also at the time of each visit, urinary NGAL (u/NGAL) was determined in a sample of the firstmorning urine previously prepared (by centrifugation at 4000 rpm) by CMIA chemiluminescent immunoassay technology (immunochemical test) (commercial kits: Abbott Diagnostic, at ARCHITECT^®^ i2000 SR analyzer. Quantitative determination was based on the microparticle principle of chemiluminescentric determination. The concentration of u/NGAL was expressed in ng/mL (upper reference limit was 131.7 ng/mL, and Limit of Quantitation (LoQ) of < 10.0 ng/mL) [25] [26].

### Statistical analysis

For statistical analysis, commercial statistical software was used: Statistica 8.0, Stat Soft Inc., Tulsa, OK, USA, 2007. All continuous variables were described as the mean ± standard deviation (x̄±SD). According to the data distribution (Kolmogorov-Smirnov test), comparisons of parametric or nonparametric continuous variables between 2 groups were performed by unpared Student's t-test or the Mann-Whitney U test, respectively. The categorical variables were expressed as percentages and examined using the Chi-square test. Spearmen's coefficient correlation tested the relationship between variables. Significance of differences was accepted at three levels of significance: (p <0.05; p <0.01; p <0.001). The sensitivity, specificity, efficiency and confidence intervals for each set of screening criteria for activity class IV-Lupus nephritis were obtained. Comparisons of receiver operating characteristic (ROC) curves were carried out to verify variations in the sensitivity and false-positive fraction (1 - specificity) of different sets of markers using overall cut-off values.

This study was conducted following the principles of the Declaration of Helsinki principles. This study was approved by the Ethics Board - MMA (f18 n74/2011).

## Results

The study groups were homogeneous in terms of basic characteristics (sex, age, body weight, LN class, therapy: Corticosteroids, Cyclophosphamide, Mycophenolate mofetil, Cyclosporine) - Table 1. Comparing the mean values of CBC elements between the groups, a statistically significant difference was observed only for the hemoglobin level (p= 0.005) initially and erythrocytes (p = 0.024) at the second visit, noting that the level was within the reference limits in both groups at all times. Monitoring of kidney function parameters (mean value of serum creatinine and glomerular filtration rate - eGFR) did not result in a statistically significant difference, which was understandable given the criteria for inclusion in the study, thus avoiding the possibility that the tested biomarker u/NGAL was a parameter for possible kidney failure. In our study, no patient had a creatinine clearance lower than 60 mL/min, which was one of the criteria for inclusion of patients in the study. In this way, we avoided the possibility that impaired kidney function affects the level of u/NGAL. In further visits, no acute increase in creatinine or development of kidney failure was observed, and values of creatinine and creatinine clearance in groups and between groups did not show a statistically significant difference thus the change in NGAL levels could only be due to the activity of lupus nephritis.

**Table 1:** Basic characteristics of participants. ^1^-Chi-square test; ^2^-unpared Student’s test

Parameters	Groups (n =20)				Comparison between groups
	Group		Group B		
Sex	female	male	female	male	p =0.40^1^
	15 (75%)	5 ( 25%)	18 (90%)	2 (10%)	
x̄ ± SD					
Age (year)	37.50 ± 15.74		42.65 ± 12.44		p = 0.25^2^
Weight (kg)<br>*Visit 0*<br>*Visit 1*<br>*Visit 2*	<br>68.74 ± 12.64<br>67.46 ± 13.60<br>66.30 ± 13,07		<br>67.27 ± 9.92<br>66.46 ± 10.53<br>65.79 ± 11.16		<br>p = 0.682^2^<br>p = 0.792^2^<br>p = 0.892^2^
Class LN	IV		IV		

A statistically significant difference between the groups was observed for total proteins and albumins in the first two visits (in Group A the level was significantly lower). Complement C3 and C4 show a statistically significantly lower value in the initial visit between groups, and anti ds DNA Ab were significantly elevated in Group A compared to Group B in all visits. The complement C3 and C4 indicated decreased disease activity to the applied therapy. Anti ds DNA Ab as well as all urinary parameters (proteinuria 24h, SLEDAI/r scor, Up/Cre, u/NGAL) show a statistically significant elevated value in Group A for all visits (p<0.001) (Table 2).

**Table 2:** Laboratory parameters monitored by visits between groups. ^*^p<0.05; ^**^p<0.01; ^***^p<0.001; ^1^-unpared Student’s test; ^2^-Mann-Whitney U test

Parameters	GROUP A<br>x̄ ± SD	GROUP B<br>x̄ ± SD	Statistical significance
Creatinine ( μmol/L)<br>*Visit 0*<br>*Visit 1*<br>*Visit 2*	<br>87.65 ± 33.09 <br>82.10 ± 27.86 <br>78.00 ± 22.59	<br>79.15 ± 19.93 <br>75.45 ± 16.03 <br>72.15 ± 16.16	<br>p=0.331^1^<br>p=0.360^1^<br>p=0.352^1^
eGFR (mL/min)<br>*Visit 0*<br>*Visit 1*<br>*Visit 2*	<br>86.52 ± 29.92<br>90.55 ± 29.73<br>94.65 ± 25.04	<br>88.61 ± 29.69<br>88.74 ± 25.88<br>94.63 ± 29.1	<br>p=0.825^1^<br>p=0.842^1^<br>p=0.997^1^
Total proteins (g/L)<br>*Visit 0*<br>*Visit 1*<br>*Visit 2*	<br>59.65 ± 9.12<br>61.80 ± 5.86<br>62.85 ± 5.20	<br>67.15 ± 9.25<br>67.90 ± 7.46<br>68.75 ± 8.46	<br>p=0.013^**1^<br>p=0.006^**1^<br>p= 0.231^1^
Albumin (g/L)<br>*Visit 0*<br>*Visit 1*<br>*Visit 2*	<br>33.35 ± 7.88<br>37.20 ± 4.25<br>38.85 ± 4.36	<br>39.05 ± 4.45<br>40.20 ± 4.67<br>40.50 ± 4.90	<br>p=0.007^**1^<br>p= 0.040^*1^<br>p=0.268^1^
Complement C3 (g/L)<br>*Visit 0*<br>*Visit 1*<br>*Visit 2*	<br>0.65 ± 0.16<br>0.5 ± 0.11<br>0.79 ± 0.09	<br>0.83 ± 0.11<br>0.82 ± 0.10<br>0.86 ± 0.12	<br>p=0.000^***1^<br>p=0.077^1^<br>p=0.062^1^
Complement C4 (g/L)<br>*Visit 0*<br>*Visit 1*<br>*Visit 2*	<br>0.09 ± 0.11<br>0.11±0.03<br>0.18 ± 0.20	<br>0.14 ± 0.03<br>0.14 ± 0.0<br>40.15 ± 0.03	<br>p=0.000^***1^<br>p=0.06^1^<br>p=0.156^1^
Anti ds DNA Ab (IU/mL)<br>*Visit 0*<br>*Visit 1*<br>*Visit 2*	<br>105.45 ± 78.03<br>90.05 ± 91.33<br>67.90 ± 80.37	<br>12.07 ± 11.71<br>12.00 ± 14.43<br>11.82 ± 31.26	<br>p=0.000^***2^<br>p=0.000^***2^<br>p=0.000^**2^
SLEDAI/r<br>*Visit 0*<br>*Visit 1*<br>*Visit 2*	<br>5.20 ± 2.66<br>2.75 ± 1.80 <br>1.60 ± 1.75	<br>0.00 ± 0.00<br>0.00 ± 0.00<br>0.00 ± 0.00	<br>p=0.000^***2^<br>p=0.000^***2^<br>p=0.000^***2^
Proteinuria 24h (g/24h)<br>*Visit 0*<br>*Visit 1*<br>*Visit 2*	<br>4.12 ± 4.08<br>2.00 ± 1.32<br>1.05 ± 0.87	<br>0.39 ± 0.35<br>0.33 ± 0.27<br>0.24 ± 0.17	<br>p=0.000^***2^<br>p=0.000^***2^<br>p=0.000^***2^
Up/Cre<br>*Visit 0*<br>*Visit 1*<br>*Visit 2*	<br>2.76 ± 2.95<br>1.38 ± 1.03<br>0.71 ± 0.507	<br>0.30 ± 0.20<br>0.26 ± 0.16<br>0.20 ± 0.07	<br>p=0.000^***2^<br>p=0.000^***2^<br>p=0.000^***2^
u/NGAL (ng/mL)<br>*Visit 0*<br>*Visit 1*<br>*Visit 2*	<br>173.25 ± 172.12<br>73.29 ± 48.76<br>49.60 ± 72.57	<br>18.75 ± 10.76<br>14.69 ± 6.27<br>13.35 ± 10.22	<br>p=0.000^***2^<br>p=0.000^***2^<br>p=0.000^***2^

In our patients with active LN class IV who were monitored and compared in visits, the mean level of u/NGAL was elevated at the initially visit (173.25 ± 172.12 ng/mL), after two months 73.2 ± 48.7 ng/mL, and in the second visit a lower level was recorded (49.60 ± 72,57 ng/mL), while the mean levels in group B were lower (initially visit: 18.75 ± 10.76 ng/mL, first visit: 14.6 ± 6.2 ng/mL, and in second visit : 13.35 ± 10.22 ng/mL). Comparison of groups yielded a statistically significantly higher level of u/NGAL in group A (p<0.001). High SD was caused by large differences in u/NGAL levels in both groups (It ranged from 28,4 to 772.3 ng/mL in Group A, and from 2,1 to 42 ng/mL in Group B).

**Figure 1:**
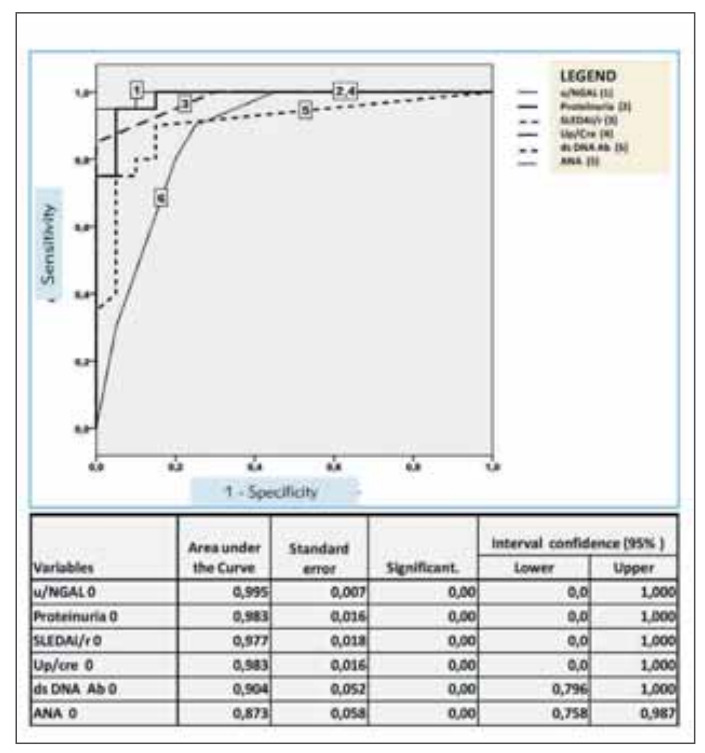
Coordinate curves for disease activity parameters and ROC curve showing Area under the Curve (AUC) of u/NGAL, proteinuria 24h, SLEDAI/r Up/k, anti ds DNA Ab, ANA.

Comparing the mean u/NGAL value between groups at 2 months with Mann-Whitney u Test we concluded within Group A, that there was a statistically significant decrease in u/NGAL, as well as SLEDAI/r score, proteinuria 24h, and Up/Cre. Having monitored changes in parameters significant for the disease activity for Group A we obtained statistical significance for albumin and urinary parameters in the first visit, and only after 4 months, in addition to albumin and urinary parameters, a statistically significant correlation was shown for other parameters. In the second visit, a negative correlation that is statistically significant for albumin and a positive correlation that is statistically significant for C3 and anti ds DNA Ab can be observed in the serum, and in the urine: a statistically significant positive correlation for SLEDAI/r, Up/Cre and proteinuria/24h (Table 3). The coordinates of the curve are presented in Figure 1. The highest sensitivity with acceptable specificity was shown by u/NGAL based on the value of the area under the curve (AUC), and the lowest sensitivity on the ROC curve was shown by ANA (Figure 1). According to the coordinates on the ROC curve for u/NGAL, the cut-off value is 52.95 ng/mL. At that value, the sensitivity is 95%, and the specificity is 100%.

**Table 3:** Correlation u/NGAL with parameters important for disease activity. ^*^p<0.05; ^**^p<0.01; ^***^p<0.001; Spearman's rho

Parameter x	Statistical<br>parametes	Parameter y					
		Albumin	C3	anti ds<br>DNA Ab	Proteinuria	SLEDAI/r	Up/Cre
NGAL<br>Visit 0	Correlation coefficient	- 0.60	- 0.33	0.26	0.94	0.54	0.93
	Probability	p<0.01	n.s.	n.s.	p<0.001	p<0.05	p<0.001
NGAL<br>Visit 1	Correlation coefficient	0.04	0.32	-0.16	0.42	- 0.06	0.41
	Probability	n.s.	n.s.	n.s.	n.s.	n.s.	n.s.
NGAL<br>Visit 2	Correlation coefficient	- 0.49	- 0.50	0.66	0.82	0.87	0.76
	Probability	p<0.05	p<0.05	p<0.001	p<0.001	p<0.001	p<0.001

## Discussion

The first studies in which NGAL was determined in patients with SLE were in the pediatric population due to the need to supplement the insufficiently instructive standard diagnosis [27] [28] [29]. Kidney biopsy, which is the standard for determining the class of LN, as well as the degree of activity and chronicity of LN is not a method that is popular in children, and the obtained data are in real-time, so the possibility of monitoring the course of treatment and adjusting the therapeutic modality is excluded. These were the reasons for the affirmation of biomarkers in lupus nephritis, and one of them is NGAL.

A study by Suzuki et al. [30], which included 85 pediatric patients with lupus nephritis, compared u/NGAL and serum NGAL (s/NGAL) levels and found that u/NGAL correlated with the renal lesion activity index, which was not the case with s/NGAL. In particular, u/NGAL levels were elevated in the group with diffuse proliferative lupus nephritis compared with membranous lupus nephritis [30]. Brunner et al. [31] described the correlation of u/NGAL with Up/Cre and a significant difference in u/NGAL levels between patients with active lupus nephritis compared to the group without active lesion as a correlation in NGAL with renal activity index SLEDAI/r, and a correlation of u/NGAL with the extrarenal index of SLE activity was not observed, which was confirmed with the results in other studies [31] [32].

In our patients with active LN class IV who were monitored and compared, the level of u/NGAL was elevated at the initially visit, after two months and four months lower level was recorded, while the levels in group B (with disease in remission) were lower. Comparison of groups yielded a statistically significantly higher level of u/NGAL in group A (p<0.001), which correlated with disease activity. We also obtained a statistically significant correlation between u/NGAL and SLEDAI/r renal activity index in Group A, but this significance persisted in the second visit despite lower levels of u/NGAL, which was explained by favorable initial treatment and the need to continue with induction therapy. When the renal activity index is 0 and the level of u/NGAL is at the level of the control group, sufficiently long follow-up of patients would indicate drug doses to be adjusted to the level of therapy to maintain stable remission. Other monitored urinary parameters: proteinuria/24h, Up/Cre were statistically significantly correlated (p <0.001) and in u/NGAL.

A group of Egyptian researchers also concluded that NGAL is a parameter that is elevated in patients with SLE who had manifested LN, as well as in those who did not manifest active LNephritis, while the control group had a statistically significantly lower level of NGAL in urine [33].

In a study by Yang et al. [33] which included not only NGAL but also IL-10, TGF-β1, and TNF-α in patients with lupus nephritis, it was concluded that TGF-β1 and TNF-α were elevated in urine and lupus nephritis but also in other autoimmune diseases, and NGAL in SLE with nephritis also positively correlated with serum creatinine, and they also concluded that u/NGAL was a highly sensitive and specific as a predictor of renal impairment compared to anti ds DNA Ab. This was confirmed by the ROC curve in which the sensitivity of u/NGAL is 0.755 to anti ds DNA Ab 0.600. Given that they did not observe a correlation with C3, C4 and SLEDAI, they considered that high levels of NGAL in the SLE group with LN (median 50.41 (199.93)) were associated with kidney impairment rather than with an active immune process [33].

In our study, by comparing biochemical and analyzes of immune response (total proteins, albumins, C3, C4) between groups, statistical significance was observed only at the beginning, which classified them into parameters with low sensitivity and specificity. It was similarly described by Brunner et al. [31] in a group of 35 patients who received a ROC sensitivity curve of 0.944 and specificity 1, where the parameters hematuria, proteinuria, complement, anti ds DNA Ab, SLEDAI/r score, Up/Cre ratio did not have comparable high sensitivity and specificity in patients with LN-confirmed biopsy. Our results were similar because we analyzed higher sensitivity and specificity of u/NGAL by analyzing the sensitivity and specificity of u/NGAL in a group of patients with active lupus nephritis and comparing them with anti-ds-DNA Ab. Analyzing the field within the ROC curve for u/NGAL, sensitivity was 0.95 and specificity 1.0, while the other monitored parameters had lower values (SLE-DAI/r score, Up/Cre, proteinuria, anti ds DNA Ab). Following and comparing the parameters of lupus nephritis activity, we noticed that u/NGAL achieved lower values much earlier in the monitored period, similar to SLEDAI/r score, Up/Cre, proteinuria, which indicated a favorable course of treatment, while antids-DNA Ab still had high titer. Monitoring of anti-ds-DNA Ab as a marker of lupus nephritis activity was, according to some authors less sensitive than u/NGAL, which was important in the range of laboratory analyses to be performed in patients with lupus nephritis and indicated the need for its routine determination in those patients [34] [35].

In the analysis of our study, we noticed that u/NGAL in comparison with the analyzes routinely used in the detection of active lupus nephritis showed high specificity and sensitivity and could represent a significant contribution to the timely detection of active disease during monitoring and treatment of those patients. Likely, its initial determination and comparison with the level in the following controls could influence changes in the therapeutic modality during the treatment of patients with LN.

*Limitations of our study*: chronic renal failure manifested before the determination of u/NGAL may have an impact on the elevated level of this biomarker, as well as the conditions of sepsis and infection. Bearing this in mind, our study did not include patients with urinary tract infection (positive urine culture), kidney calculus,and with kidney failure (CKD eGFR < 60 mL/min/1.73 m^2^)

## Conclusions

The results of our study indicate that the level of uNGLA is elevated in patients with active Lupus nephritis class IV, as well as that it correlates with other parameters of disease activity. Serial determination of u/NGAL could be significant in monitoring disease course and treatment.

## Dodatak

### Authors’ contributions

All authors read and approved the final version of the manuscript.

### Conflict of interest statement

All the authors declare that they have no conflict of interest in this work.
